# Influence of Different Measuring Backgrounds on the Classification of Multilayer Polyolefin Films Using a Near-Infrared Handheld Spectrometer

**DOI:** 10.1177/00037028241307034

**Published:** 2024-12-26

**Authors:** Hana Stipanovic, Patrick Arth, Gerald Koinig, Nikolai Kuhn, Jakob Lederer, Dominik Blasenbauer, Anna-Maria Lipp, Alexia Tischberger-Aldrian

**Affiliations:** 1 Chair of Waste Processing Technology and Waste Management, Department of Environmental and Energy Process Engineering, 111504 Montanuniversitaet Leoben, Leoben, Austria; 2 Christian Doppler Laboratory for a Recycling-Based Circular Economy, Institute of Chemical, Environmental and Bioscience Engineering (ICEBE), 27259 TU Wien, Vienna, Austria

**Keywords:** Near-infrared, NIR, handheld, spectrometry, multilayer plastic films, measurement background, polyolefin classification method, transflection, plastic waste

## Abstract

The low thickness of plastic films poses a challenge when using near-infrared (NIR) spectroscopy as it affects the spectral quality and classification. This research focuses on offering a solution to the challenge of classifying multilayer plastic film materials with a focus on polyolefin multilayer plastics. It presents the importance of spectral quality on accurate classification. The aim is to demonstrate the suitability of the handheld NIR spectrometer in classifying multilayer polyolefin films and assess the impact of various measuring backgrounds (white tile, Teflon, aluminum, copper, silver, and gold) on classification accuracy in the wavelength range of 1596–2396 nm. Metallic backgrounds have been found to enhance spectral quality and classification accuracy. The classification accuracy was consistently high, ranging from 96.55% to 100%, with aluminum and gold backgrounds yielding the best results in theoretical accuracy. In experimental classification, the accuracy reached 100% when any metallic backgrounds were used. Conversely, Teflon showed a theoretically high accuracy of 96.21% but only achieved an experimental accuracy of 72.2%. These findings suggest that using metallic backgrounds can improve the spectral quality and classification of plastics with low thickness (films) and complex material composition (multilayers).

## Graphical abstract

**Graphical abstract:**
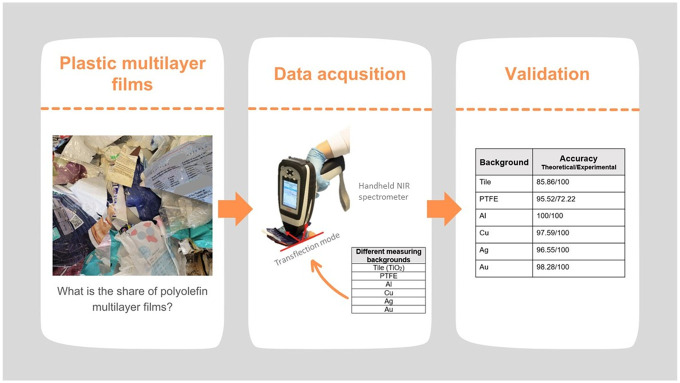
This is a visual representation of the abstract.

## Introduction

Plastic waste and its handling are some of our generation's biggest challenges. Near-infrared (NIR) spectroscopy has proven to be the state-of-the-art technology for plastic waste classification and separation. NIR light is part of the electromagnetic spectrum and lies in the range of 0.7–3 µm (wavenumber 12 000–4000 cm^–1^), near the range of visible light (0.4–0.7 µm).^1^ Material identification using NIR spectroscopy is achieved by comparing the obtained spectral information with a library of credible reference spectra, forming material-specific characteristic spectra. Furthermore, a single spectrum enables the simultaneous determination of several analyses, as well as the determination of nonchemical (physical) parameters such as density, viscosity, and particle size in pulverulent solids.^2–4^ The most significant gain of NIR spectroscopy is in analyses where a considerable number of samples are used.^5^ Introducing portable NIR devices has significantly improved performance over existing benchtop systems by increasing operation speed, portability, and ruggedness while reducing power consumption, size, and weight.^3^

This study presents an application of a handheld NIR spectrometer for classifying multilayer polyolefin films and examines the influence of different measuring backgrounds on classification.

European plastic production reached 58.7 Mt in 2022 after stagnating in 2020 due to the COVID-19 pandemic.^6^ The success of plastics is mainly due to their comparatively low cost, low density, and numerous mechanical and thermal properties, which make them suitable for a wide range of applications.^7^ One of the most important applications is the packaging of goods, accounting for 39% of all plastics produced in 2022.^6^ Polyolefins, such as polyethylene (PE) and polypropylene (PP), are the most common plastics in packaging, accounting for nearly 75% of packaging by weight. Polyolefins are extremely versatile polymers with excellent properties that make them ideal for the production of flexible packaging films.^8^ Flexible plastic films are a popular choice for packaging mainly because of cost efficiency and versatility. They are lightweight, reducing shipping and production costs as they require less material, making them more economical for manufacturers and consumers. They can also be adapted to fit different shapes and sizes, making them suitable for a wide range of products.^9,10^ Still, most packaging, especially film packaging, is often discarded after a single use, leading to a significant amount of plastic waste. In 2020, the European Union collected approximately 29.5 Mt of post-consumer plastic waste, with packaging waste accounting for 17.9 Mt of this total.^11^

Packaging generally consists of a single material. However, increasing demands on packaging have necessitated the development of advanced packaging techniques. Subsequently, this has led to rising amounts of multilayer plastic materials that are better suited to fulfilling the properties required of packaging than monolayer plastic materials. A multilayer plastic structure consists of different plastic materials used to combine the respective performance of different polymers, allowing these multilayers to combine functions that would be impossible with one layer. Each layer in a multilayer film serves a specific purpose, enhancing the overall performance of the material, e.g., moisture and gas barriers, durability and flexibility, material optimization, heat sealability, printability, appearance, and surface properties. Usually, the inner layer provides sealability, and the outer layer resistance against abrasion, printability, or barrier. Eventually, the layers can also be thinner, so less material is needed than for a single layer of plastic to fulfill the same function, which improves the product-to-packaging weight ratio.^12^

While their properties, such as low weight and ability to perform various packaging functions by combining different polymers make multilayer materials a complete packaging solution, the challenges of sorting and recycling remain.

According to Koinig et al., in the separately collected waste in Austria, 30 wt% is flexible two-dimensional (2D) plastic packaging, of which 20 wt% are multilayer films. At its meeting in December 2020, the European Council welcomed the Commission's intention to ensure that all packaging is reusable or recyclable in an economically viable way by 2030.^13,14^

This decision puts pressure on manufacturers to redesign multilayer packaging to be recyclable. Polyolefins have strong potential to replace other polymers used in multilayer packaging materials, but they still must meet specific performance criteria in order to do so. The main challenge is the barrier properties, which do not yet provide adequate moisture and gas barrier properties.^15^ Additionally, they have to achieve the mechanical strength, heat sealability, and surface properties of other polymers used for these purposes while also making them economically viable.^16^ The recyclability and redesign of multilayer food packaging were analyzed by Baier et al., and it was concluded that, in the future, polyolefins should be the remaining materials used for multilayer packaging design.^17^ Therefore, significant research is being conducted to gain the desired properties to reach this goal.

Considering the significant use of polyolefins in various applications, different spectroscopy methods have been developed to characterize and classify waste polyolefins. Neo et al.^18^ and Adarsh et al. published review studies in 2022 on the application of spectroscopic methods to plastic waste.^19^ IR spectroscopy is the most widely applied spectroscopic method for plastic waste classification. With wavelength ranges of 700–2500 nm for NIR spectroscopy and mid-infrared (MIR) with wavelength ranges of 2500–25 000 nm, two regions of IR are primarily used in developing various classification methods for plastic waste.

Spectroscopic techniques generally offer the advantages of minimal to no sample preparation and eliminate the need for reagents or chemicals, offering the possibility of acquiring qualitative and quantitative results. Besides, NIR spectroscopy's primary advantages are the high speed of analysis, its cost-effective instrumentation, robustness, diverse portability options, and subsequently, in combination with data analysis techniques, the possibility of the automatic in-line and on-line assessment coupled to a sorting machine.^19^ Hence, it has become the most used method for sorting plastic packaging waste.^18^ Despite its many advantages, one disadvantage is that the packaging is often black. This presents a challenge because carbon black is strongly absorbed in the NIR region, resulting in featureless spectra.^20^

Mid-infrared spectroscopy is an alternative to NIR spectroscopy for classification purposes, as it is less affected by the color of plastics and can even classify black plastics.^21^ However, MIR spectroscopy has higher instrumental costs, lower spectral acquisition speed, a shorter operational lifetime, and limited penetration depth, making it more suitable for surface analysis.^19,22,23^ It is mainly used in laboratory environments due to its sensitivity and high costs, often as a reference for developing other spectroscopic methods.^24^ In this case, the Fourier transform infrared spectroscopy (FT-IR) instruments are predominantly used, offering high-resolution spectral data over a wide range and a high signal-to-noise ratio with high spectral resolution.^21^ Raman spectroscopy does offer advantages over NIR spectroscopy. It complements MIR spectroscopy but is not used as frequently due to complications with background fluorescence, which can often overshadow certain peaks of interest.^18,25^ Laser-induced breakdown spectroscopy (LIBS) allows qualitative and quantitative analyses to be conducted simultaneously. It is still relatively new, yet it is still used in laboratory settings due to its calibration challenges and sensitivity to environmental influences.^26,27^ Fluorescence spectroscopy is a non-destructive method capable of identifying additives in plastics and differentiating between types of plastic.^28^ Yet it faces various problems, such as loss of signal over time, known as photobleaching, and sensitivity to environmental factors.^29^

Although almost all spectroscopic methods have been proven to classify polyolefin plastic waste successfully, NIR spectroscopy still stands out with its advantages and high level of research. Only NIR spectroscopy was used in studies on the classification of multilayer plastics, while the other methods were not represented.^30^ However, the identification of thin materials, like plastic films, can present an additional challenge. The utilization of reflective materials as measuring background enhances the likelihood of successful detection when separating films by improving the signal's intensity and clarity. Its reflective properties help to increase the contrast between the sample and the background, thus supporting more accurate identification.^31^

The reflective materials can reflect the NIR light, preventing it from being transmitted.^32^ The reflective background enables a second passthrough of the NIR radiation, decreasing the amount of radiation lost to transmission and increasing the likelihood of interaction between the radiation and the sample's molecules. In order to obtain spectra with a high information content, sufficient interaction of the rays with the matter is required. The measurements in transflection mode strongly expand the range of samples that can be measured, including plastic films.^33^

Plastic waste is primarily mechanically recycled. However, not all plastic materials can be easily recycled using state-of-the-art mechanical recycling processes. Recycling of flexible plastic films presents several physical and logistical challenges that can limit the efficiency of the recycling process. They present a challenge for the shredding step since they tend to wrap around the shredder blades, clogging the system. Thin films can also have inconsistent melting behaviors because of different molecular weights, leading to lower-quality recycled material. Additionally, their low mass-to-volume ratio can make their handling and transportation complex.^10,34,35^ Recycling of multilayer plastic films adds another level of complexity to it, with the need to separate different components (polymers) before further processing.^12^

In addition, the mechanical recycling of polyolefins faces particular challenges. Its primary disadvantage is that the process degrades the mechanical properties of polyolefins, which means that the plastic is “down-cycled” into secondary and less valuable products.^36^ Regulations also prevent mechanically recycled polyolefins from being used in food contact applications. Multilayer plastics, especially polyolefins, are, therefore, attractive waste streams for chemical recycling, which is presented as a key solution, particularly for complex polyolefin waste streams.^37,38^

Chemical recycling converts plastic into valuable feedstock from which virgin-like plastics can be produced without any loss of quality or restrictions on the type of applications.^36^ Additionally, there is also the possibility of recycling mixed polyolefin waste, including multilayer packaging. The development of chemical recycling has created complementary solutions to existing mechanical recycling for waste that would otherwise be incinerated.^39^ Despite its advantages, one of the main drawbacks of chemical recycling is the need for high-purity input material, which is the key to yield. The quality of the input material and the subsequent sorting steps significantly influence achieving a high yield.^36^ It is crucial to quickly and reliably verify the purity of the delivered input material without high costs and time-consuming laboratory analyses. The handheld NIR spectrometer can support on-line NIR monitoring tools by verifying the composition of representative samples taken during operation. It is also an additional tool for the validation of automated NIR monitoring methods.

The use of a handheld NIR spectrometer for the classification of diverse plastic monolayer materials (simple measurements) has already been researched by various authors like Rani et al. and Yang et al.^40,41^ In contrast, the clear identification of multilayer plastic materials is more complex, especially if the sample is only to be measured from one side, which has not yet been researched. Furthermore, the low material thickness of films poses an additional challenge as it leads to lower spectral quality, which is crucial for sufficient classification. The research presented by Masoumi et al. has shown that increasing material thickness leads to more pronounced spectra since differences in spectral curves can be more easily identified with high reflectivity and spectral information increases.^42^ Koinig et al. investigated the methods for capturing valuable spectra even from very thin plastic materials.^31^ They evaluated the influence of different reflective materials on the NIR spectra of the films in the wavelength range of 911–1677 nm by placing reflectors made of copper or aluminum behind the material, which enabled detection in transflection rather than reflection mode. Finally, it was concluded that the spectral variability decreased considerably when using a reflector made of copper or aluminum as a background for the benchtop NIR spectrometer.

This study aims to show that the handheld NIR spectrometer offers the possibility to classify multilayer polyolefin films and how different measuring backgrounds affect the classification in the 1596–2396 nm wavelength range. This study focuses not only on the application of copper and aluminum as backgrounds, but also on other metallic backgrounds, such as gold and silver. In addition, Teflon (polytetrafluoroethylene, or PTFE) as another material with high reflectivity in the NIR spectrum and white tile as a reference background, were examined. Together with copper and aluminum, gold and silver are proven to have a high reflectivity in the NIR wavelength range.^32^ Reflectivity is an optical property of the material, which refers to its ability to reflect incident light or electromagnetic radiation. In our case, the reflectivity of the substrates is defined by the reflectance efficiency within the NIR wavelength, typically measured as a percentage of the total energy.^43^ Silver has the highest IR reflectance over an extended wavelength range of any known material, reaching up to 99%,^44^ aluminium reaches up to 90%, copper up to 95%, and gold between 95% and 98%.^32^ On the other side, Teflon has the unique property of having almost constant reflection over the wavelength range of 250–2500 nm.^45^

The objectives of this study are the following: (i) Introducing a method for detecting multilayer polyolefin films using a handheld NIR spectrometer. (ii) To enable the detection (differentiation) of multilayer polyolefin and non-polyolefin films with only one measurement while measuring from only one side of the sample. (iii) The influence of applying various measuring backgrounds on the detection performance is examined during the development of the method. (iv) Following the investigation of the influence of different measuring backgrounds, a recommendation is made on the usage of certain measuring backgrounds.

**Figure 1.**
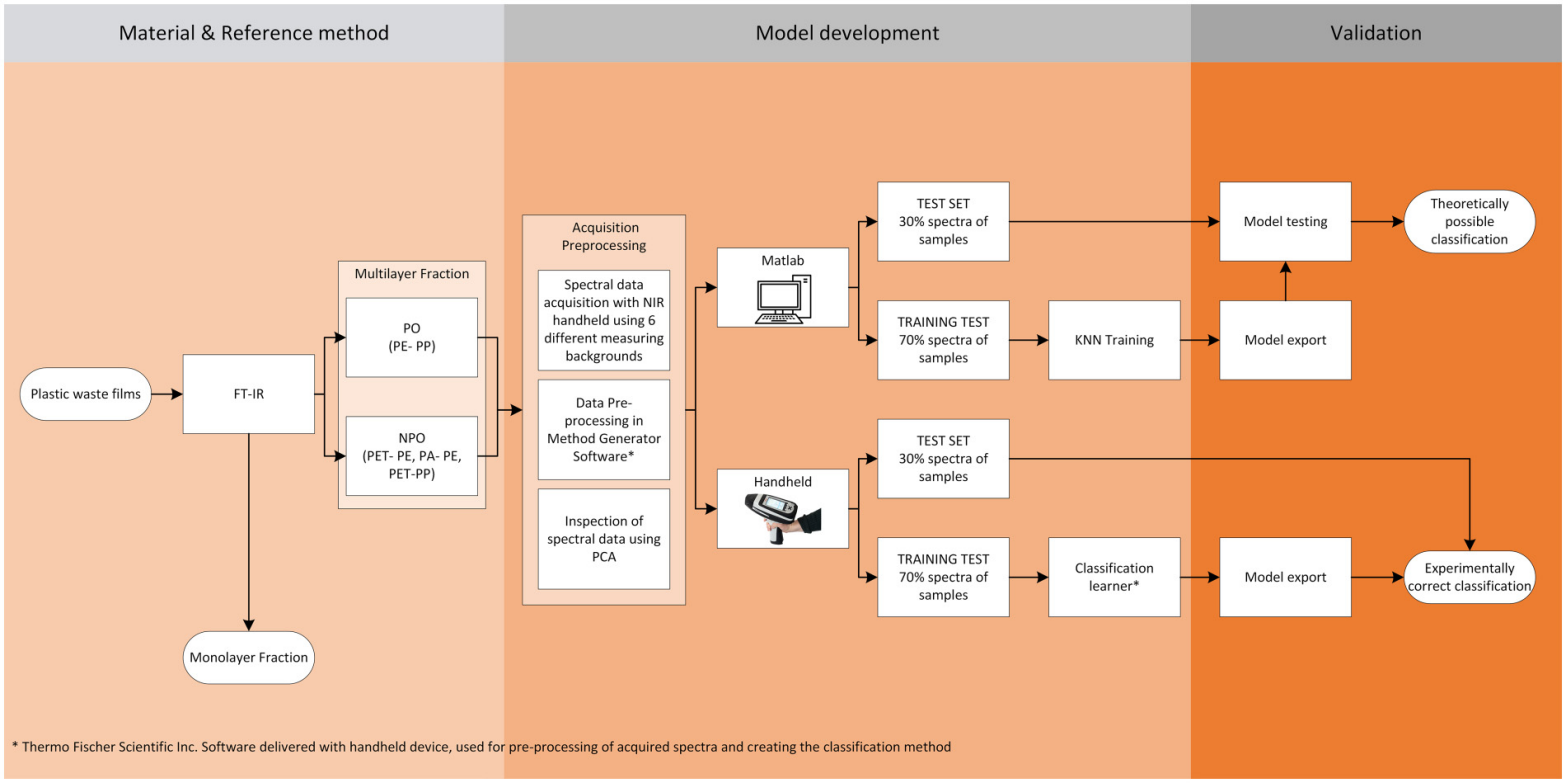
Schematically presented structure of the experiment.

The entire structure of the experimental part of the study is presented in Figure 1.

## Experimental

### Materials and Methods

The material used for the measurements was a 2D plastic packaging fraction (films) originating from the eject stream of the NIR sorting step of a material recovery facility (MRF) in Austria. The eject fraction was a light fraction from the wind sifter, which was then subjected to NIR sorting to remove plastics (except PVC, or polyvinylchloride) and polyurethane (PU) from the stream. The collected samples were then sorted by hand to obtain the film fraction of the samples. The entire sampling procedure is explained in detail in Blasenbauer et al.^46^

A total of 184 samples of plastic packaging films were analyzed. The samples were used in their original state without washing or shredding steps, whereby colored and transparent samples were used. The size of the samples varied from 62 to 207 µm in thickness and 4–20 cm in width and length.

### Reference Method

All samples used were analyzed with Agilent Technologies’ Cary 360 FT-IR spectrometer as a reference method, as it has an established spectral database, high resolution and high sensitivity. FT-IR spectrometer performs measurements in the wavenumber range of 4000–400 cm^–1^ and is mainly used to estimate the structure of materials based on the specific peaks related to different functional groups.^47,48^ This technique is based on the identification of functional groups in molecules that vibrate when irradiated with specific wavelengths of light. These vibrations and their intensity are plotted against the frequency of light (cm^–1^) to which the sample is exposed to produce an FT-IR spectrum. The acquired spectra are then compared to previously recorded and stored spectra of different plastic materials.^49^

The spectra were collected in attenuated total reflection (ATR) mode, resulting in ATR FT-IR measurements and samples spectra. The Polymers and Polymer Additives P/N, 30002 spectrometer database provided by Agilent was used to identify collected samples. The database consists of pre-recorded spectra of reference plastic materials. It contains over 5000 spectra of mainly pure materials and compounds that cover the plastics typically in the 2D plastic waste fraction. Due to the limited penetration depth of FT-IR, which typically identifies only the outermost layer of multilayer plastic films, measurements were performed on both sides of the sample to distinguish single-layer from multilayer plastic samples. This approach served as the initial step in the sample separation process. The entire multilayer fraction, consisting of 184 samples, was used in the experiment.

**Figure 2.**
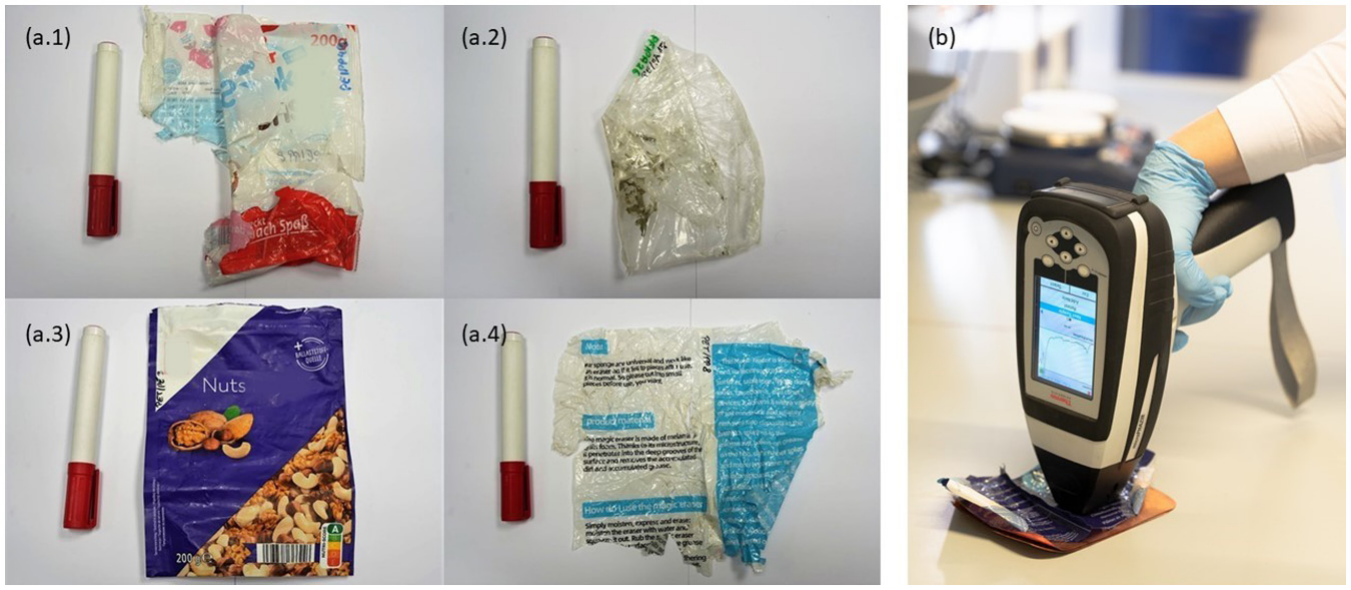
Examples of samples from different fractions used in the experiments: (a.1) PE-PP (PO), (a.2) PE-PA (NPO), (a.3) PET-PE (NPO), (a.4) PET-PP (NPO), and (b) an example of performing measurements with the microPHAZIR handheld spectrometer with copper as measuring background.

The samples were analyzed to define their composition. Four fractions of plastic multilayer films were identified: PP-PE, PET (polyethylene–terephthalate)-PE, PA (polyamide)-PE, and PET-PP, which only applies to the outermost two layers. Furthermore, multilayer films with the composition PP-PE were assigned to the polyolefin (PO) fraction and the others (PET-PE, PA-PE, and PET-PP) to the non-polyolefin (NPO) fraction. The examples of one sample from each identified fraction are shown in Figure 2a, the PO (PP-PE) fraction in Figure 2a.1, the NPO fractions, PE-PA in Figure 2a.2, PET-PE in Figure 2a.3, and PET-PP in Figure 2a.4. Finally, the PO fraction consisted of 58 samples and the NPO fraction of 126 samples. In order to be able to carry out the method development and validation of the methods developed, the samples were additionally split into a training set (70% of samples) and a test set (30% of samples), both of which equally represent the groups of samples used in order to make the validation reliable. The training set of samples was exclusively utilized for method development, while the test set was reserved for validating the developed methods. The test set consisted of samples not previously used in the training phase, ensuring the methods were evaluated on unseen data, thus providing an objective assessment of their performance.

### Model Development

The spectral acquisition of training and test sample sets of samples was performed with the microPhazir NIR spectrometer (Thermo Fisher Scientific Inc.). The spectrometer was developed to perform diffuse reflection measurements in the 1596–2396 nm (6250–4100 cm^–1^) spectral range and is among the most known NIR handheld spectrometers due to being lightweight and user-friendly, as well as offering rapid analysis within seconds, making it a popular choice for field applications. The microPhazir was introduced to the market about 10 years ago, and although many others have been introduced to the market since, some even with higher resolution and broader wavelength ranges, the microPhazir remains one of the most utilized ones.^50^ The microPhazir offers comparable wavelength ranges to other NIR handheld spectrometers but tends to be more application-specific with wavelength range focused on higher NIR wavelength region. The spectrometer's wavelength range includes the end of the first overtone region, which is important for detecting vibrational modes of C–H, O–H, and N–H bonds. These bonds are significant for the identification and analysis of materials like plastics, where these bonds are the main part of chemical structure. The higher wavelength within the NIR spectrum results in higher intensity of the vibrations, enabling distinguishing complex organic substances. Hence, the observed range includes higher overtones, leading to a higher signal response and more pronounced peaks compared to the lower wavelengths while capturing combination bands and second overtones. However, certain bands within higher wavelength range may overlap due to overtones and combinations modes impeding the assignment of the NIR bands.^51–53^

The device is equipped with a light source for the NIR radiation, a wavelength selector, a sample chamber that brings NIR radiation into contact with the sample, and a detector that measures the interaction between the sample and the NIR radiation.^54^ In the case of microPhazir, the light source is a tungsten–halogen lamp with a MEMS spatial light modulator and a single-element indium–gallium–arsenide (InGaAs) detector. Scanning and generating a complete spectrum of the sample is done in less than 10 s with satisfyingly good resolution. The speed, the simplicity of the measurements and the fact that there are no moving parts make the device suitable for on-site measurements.

The measurements were performed in transflection mode using different measuring backgrounds to improve the signal quality and, thus, the classification. The materials used as measuring backgrounds were white tile, Teflon, aluminum, copper, gold, and silver. A regular glazed ceramic tile with a white and even surface was used instead of an expensive reflectance standard for the white tile background. Teflon background was a PTFE disk used to conduct chemical reactions in the laboratory microwave. The metallic backgrounds investigated were copper plates with 99.9% copper, rolled 100% aluminum, and pure silver and gold leaf. Due to their low thickness and easy breakage, the silver and golden leaves were fixed and protected with glass on the top, and black plastic on the back. Glass was chosen because it has no significant spectral features in the NIR region.^55^ Black plastic was chosen because carbon black absorbs strongly in the NIR region and, therefore, should not affect the reflectivity of metals.^56^ All other backgrounds were used in their original state. The sizes of backgrounds were all in the range of approximately 10 × 10 cm. An example of a measurement carried out with a copper background is shown in Figure 2b.

The samples were each positioned on the selected background, and the spectrometer was placed directly on the sample. All the samples were measured from only one side of the samples (simple measurement) in five different positions, acquiring five spectra per sample, resulting in a total of 920 raw spectra (290 PO and 630 NPO spectra).

The acquired spectra of the samples require signal pre-treatment to be used for further analysis. After data collection, pre-processing the spectral data prior to chemometric modelling is essential, mainly because the spectra can be significantly influenced by non-linearities introduced by light scatter. With suitable pre-processing, these effects can be largely eliminated. The most commonly used pre-processing techniques can be divided into scatter-correction methods and spectral derivatives.^57^ Various pre-processing tools were applied to the raw data and used to pre-process the data prior to developing the classification methods. Various combinations of scatter-correction methods, including MSC and SNV, along with spectral derivatives, such as the first and second derivatives, with the application of the Savitzky–Golay filter, were evaluated. A combination of applying the Savitzky–Golay five-point, second-derivate procedure with a second-order polynomial followed by standard normal variate (SNV) gave the best results in extracting absorption bands, removing instrument noise and spectral data refinement, and eventually the highest classification accuracy.

The Savitzky–Golay filter is based on the reduction of instrumental noise of the reflectance spectra and the subsequent extraction of absorption band positions (wavelengths) using high-order derivatives.^58^ Combined with the second derivative, it can sharpen peaks and reduce baseline drift, making resolving overlapping bands in NIR spectra easier. Additionally, the second derivative Savitzky–Golay, enhances multivariate analysis by making subtle spectral differences more apparent, essential for improving model accuracy.^59^

Standard normal variate is part of the pre-processing methods for scatter correction that effectively removes the multiplicative interference of the scatter. In this method, each spectrum is centered and then scaled by the corresponding standard deviation.^60^ This process improves the consistency of the spectral data while making it more suitable for further analysis.

The acquired spectra underwent pre-processing steps before being used in further classification. The classification was performed in two levels, theoretical classification using principal component analysis (PCA) and *k*-nearest neighbor (KNN) classification for each used background using Matlab v.R2022b with chemometric toolboxes, and experimental classification using the Method Generator software, software delivered with the spectrometer, and a handheld NIR spectrometer itself. Matlab was used for comprehensive analysis of the acquired data, employing PCA and KNN classification for each evaluated measuring background to guide the on-board method. The Method Generator software was used to develop methods using all six different measuring backgrounds, which were then tested using a handheld NIR spectrometer to gain experimental results. The methods were developed using dimensionality reduction and KNN classification. Theoretical results, derived from the same framework, dimensionality reduction (PCA) and KNN, are thus directly comparable to the experimentally obtained classification.

In the first step, the acquired spectra of the training set samples were used to perform dimensionality reduction with PCA using Matlab to determine the theoretical separation potential of the multilayer films.

Principal component analysis is an essential and powerful method in chemometrics.^61^ The core of PCA is to find new uncorrelated variables that are linear combinations of the original dataset while retaining as much statistical information as possible.^62^ PCA aims to extract the information encoded in a certain number of variables into a smaller set of new orthogonal variables called principal components (PC).^40^ The PCs are created in order to preserve as much variability as possible. They are new variables that are linear functions of the variables in the original dataset that successively maximize variance and are not correlated with each other.^63^ The method was applied to the training set of data, providing an overview of complex multivariate data, examining the data structure, reducing the dimensionality and extracting the data information. It was also used as the first prediction of classification capability for multilayer polyolefin films using a handheld NIR spectrometer. The results obtained after performing PCA were used to create a classification model as a validation tool based on the KNN algorithm to obtain theoretically possible classification.

Conversely, classification methods were developed for each analyzed background to obtain experimentally correct classification. The methods were developed using the Method Generator software. The methods developed demonstrated the handheld NIR spectrometer's potential for classifying polyolefin multilayer films in the experimental setup. The method was developed using reference data based on the training set of samples, using a classification learner that performs spectral matching using KNN prior to model export onto the handheld.

## Validation

### Theoretically Possible Classification

After performing the PCA, all the scores obtained were used as a basis for building a classification model using a supervised classification tool. The classification tool used was KNN, currently the most widely used supervised classification algorithm.^64^ KNN classifies unlabeled data by calculating the distance between each unlabeled data point and the nearest neighbors of the new data point. It then assigns each unlabeled data point to the most identically labeled data class by finding patterns in the data set.^65^ The classification is based on the KNNs (smallest distance), where *k* is the number of neighbors involved in most voting processes.^66^

Since the classification depends on the number of nearest neighbors involved, the classification was performed with up to 10 different neighbors. The KNN classification was performed with Matlab with both training and test data sets, using the training data set to vote on the class label of the new data. In contrast, the test data, representing previously unseen information, was utilized to evaluate the performance of the developed model. This allowed accuracy levels to be determined for different numbers of neighbors. The developed model was exported and tested, and the results obtained were used as a further theoretical argument for the possible classification of polyolefin multilayer films, presenting theoretically possible classifications.

### Experimentally Correct Classification

The methods developed for the handheld NIR spectrometer were developed using reference data based on the training set of measurements performed with all measuring backgrounds, where measurements with one background were used to develop one method, resulting in six different methods. The methods were developed using the associated Method Generator software, with the data going through the same pre-processing steps as in theoretical data evaluation for theoretically possible classification: pre-processing, size reduction, and KNN classification. The developed methods were then validated using a test set of samples. The obtained results were used to demonstrate the experimental possibility of using a handheld NIR spectrometer to classify polyolefin multilayer films, resulting in an experimentally correct classification.

## Results and Discussion

### Pre-Processing

**Figure 3.**
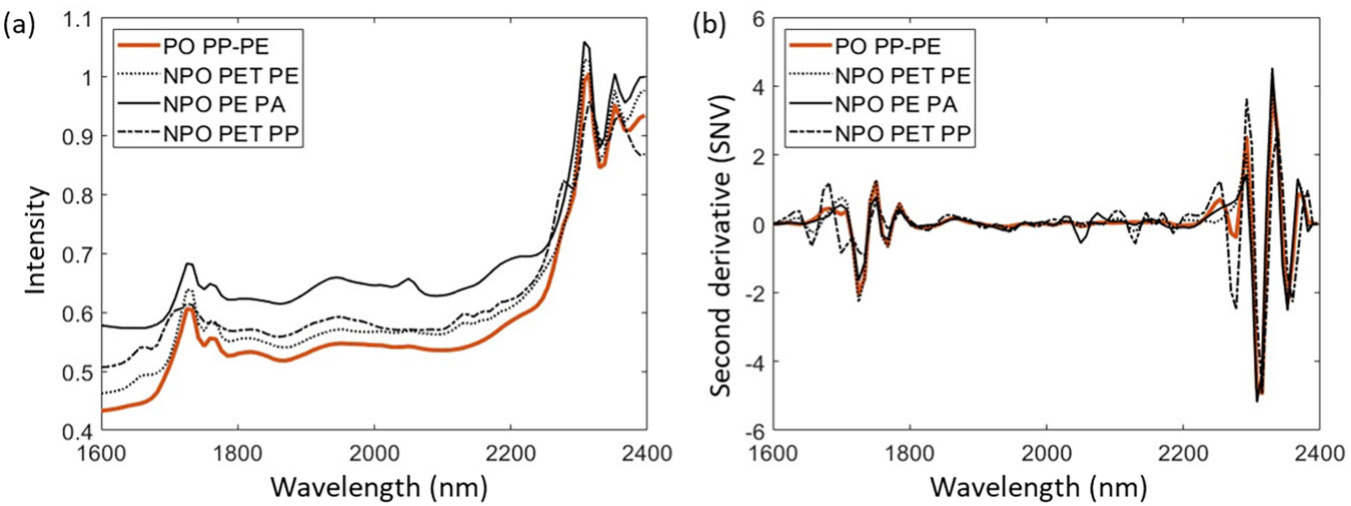
Near-infrared (NIR) spectra of (a) raw and (b) pre-processed mean spectra of polyolefin and non-polyolefin samples on the example of gold background.

The absorbance spectra of the mean spectra of four analyzed multilayer fractions (one PO and three NPO) with a focus on the PO (PE-PP) fraction are shown in Figure 3. Figure 3a shows raw mean spectra. Figure 3b shows corresponding spectra after applying pre-processing steps using the Savitzky–Golay second derivative and SNV.

In the raw spectrum of PO shown, the characteristic peaks of PO spectra can be seen in the wavelength ranges of 1700–1800 nm and 2250–2350 nm. The spectra of NPO samples show characteristic peaks in the same areas, with some less pronounced peaks in the other wavelengths.

The less prominent peaks become more pronounced following pre-processing steps, thereby enhancing the ability to differentiate between the spectra of PO and NPO samples. The spectra of PO maintain their characteristic peaks, which become more pronounced after pre-processing. In contrast, the spectra of NPO exhibit additional characteristic peaks that are more prominent compared to the raw spectra. The peaks mentioned are found at wavelength ranges of 1630–1700 nm and 2000–2250 nm, which makes it possible to distinguish between PO and NPO spectra.

### Principal Component Analysis (PCA)

In order to achieve dimension reduction, explain the variations in the data set, obtain the most important data for further classification, and finally illustrate the comparisons, PCA was performed using Matlab for complete analysis of samples measured using each background. PCA was used to explore the theoretical possibility of separating PO and NPO sample fractions with different measuring backgrounds. After the spectral acquisition, the PCA extracted the information into a new set of variables, principal components (PC), which were used as a basis for further theoretically possible KNN classification steps.

Each PC explains a portion of the total variance in the data, with the first PC containing most of the explained variance, while each following PC explains less variation. The first PC represents the most prominent features in the spectra, while each subsequent PC represents an additional, less dominant pattern in the data. The acquired PCs can be analyzed keeping this in mind. Using reflective surfaces that prioritize the larger percentage of variance in the PC1 ensures that the most significant characteristics of the data are compiled, which is particularly important given the volume of data often being processed. For all metallic backgrounds and white tile, the first PC already explains more than 70% of the variance, indicating that the most significant features of the spectra are recognized and pointing out the high probability of the separation between the fractions. In contrast, the first PC of a Teflon background explains only 46% of the variance, indicating the lower possibility of successful separation between the fractions. Subsequently, the other PCs follow this pattern, with the sum of the first four PCs of metallic backgrounds and white tile explaining more than 95% of the variance and Teflon only 74%.

**Figure 4.**
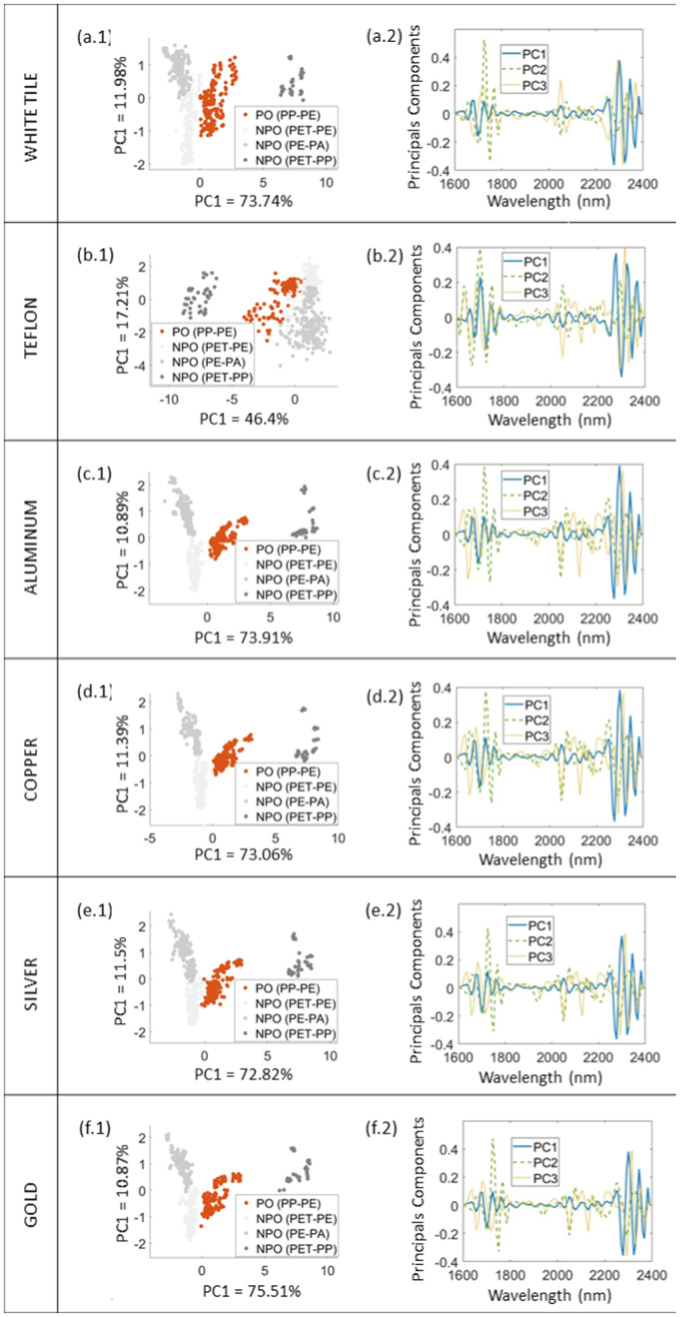
Score plots of PCA (PC1 versus PC2) together with loading plots for (a) white tile, (b) Teflon, (c) aluminum, (d) copper, (e) silver, and (f) gold.

While the PCs represent a new dimension to maximize the variance, loadings of PCA designate the contribution of original variables to the PCs, and the scores represent the data in the transformed space. To better understand the results obtained, the score plots are shown in Figure 4 together with loading plots for the first two principal components (PC1 versus PC2) of all backgrounds analyzed, that is, white tile (Figure 4a), Teflon (Figure 4b), aluminum (Figure 4c), copper (Figure 4d), silver (Figure 4e), and gold (Figure 4f). The score plots show the first two PCs, accounting for over 80% of the explained variance in all analyzed backgrounds except Teflon.

Each cluster represents a group of samples, with those in close proximity having similar spectral characteristics, either PO or NPO. Since the NPO fraction consists of three multilayer plastic fractions, the PO cluster is found in between those. On the left side of score plots, the two NPO fractions containing PE (PA-PE and PET-PE) are found, while the NPO fraction containing PP (PET-PP) is to be found on the right side. The PO (PE-PP) fraction is separated from the NPO fractions in all score plots, indicating that the different fractions can be discriminated using PCA based on the NIR spectra.

As mentioned above, the first four PCs of white tile and metallic backgrounds account for almost the same percentage of explained variance, while Teflon accounts for 20% less. Although PC2 and PC3 can still contain valuable information and contribute to classification accuracy, the primary objective is to capture as much relevant variance as possible within PC1. By doing so, the amount of data required for classification is reduced, allowing to focus on the wavelengths containing most information. The key goal is to minimize the data volume while still containing the most useful information, particularly for further sensor-based sorting applications. In such cases, reducing the spectral range that needs to be analyzed would lead to shorter measurement times, resulting in higher speed and accuracy.^67^ When analyzing the score plot of Teflon, it is possible to recognize that some of the PO and NPO data overlap, in contrast, to score plots of the metallic backgrounds where that is not recognizable. This also suggests that classification using metallic backgrounds would theoretically require fewer PCs. In the case of distinguishing PO from NPO, PC1 alone could be sufficient, whereas classification using Teflon would necessitate incorporating more data into the process.

Although the tile and metallic backgrounds have almost the same share of explained variance, it is still possible to identify different behaviors of the collected data. The data from the metallic backgrounds show improved clustering, as evidenced by the confidence ellipse analysis. A smaller confidence ellipse reflects this in comparison to the one from the white tile. This suggests lower variance, a more compact distribution of data points, and better spectral quality of the samples measured using metallic backgrounds than non-metallic ones.

In addition, slight differences can be observed in the distinct clustering within the score plots of metallic backgrounds. Analysis of the confidence ellipses reveals that aluminum and copper exhibit slightly smaller ellipses than silver and gold. However, the first two principal components of gold account for more explained variance than those of other metallic backgrounds. Surprisingly, silver shows the worst data clustering despite its high reflectivity rate, which corresponds to the lowest explained variance of data within metallic backgrounds. This may be because silver corrodes in ambient atmospheres, which may affect the reflectivity of the material.^68^

**Figure 5.**
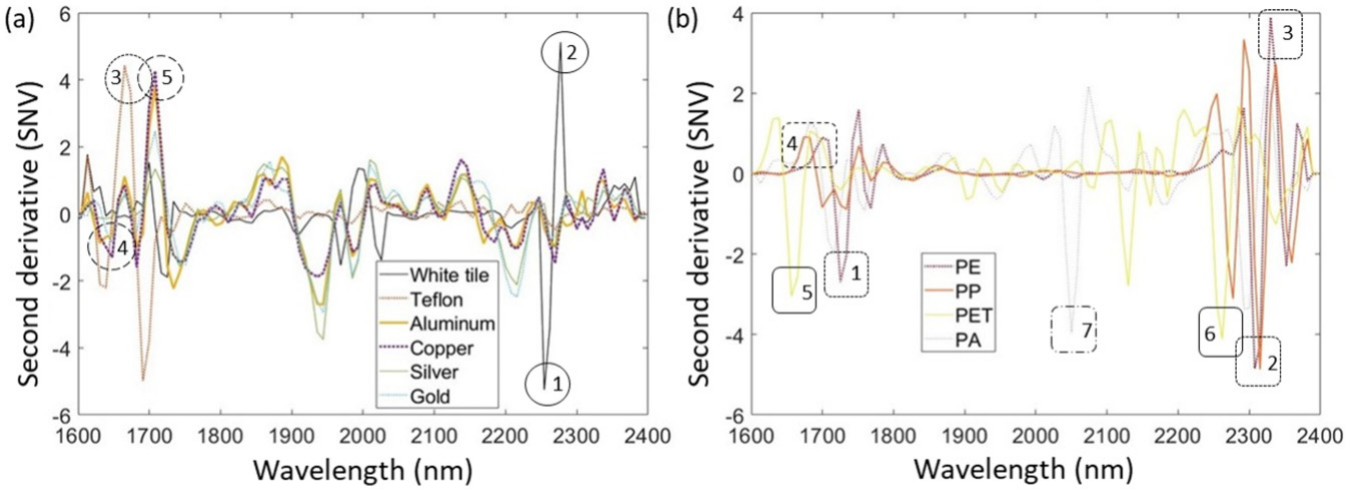
Pre-processed reference spectra of (a) used backgrounds and (b) single-layer plastic materials along with the specific peaks marked for each background and plastic material.

Furthermore, loading plots were presented to understand the contribution of different influences such as background or material composition. The loading plots show the wavelengths that contribute significantly to a particular principal component and help to identify key spectra features. The loading plots for samples measured with each analyzed background consisted of the first three principal components since they account for most of the variance. Figure 5 shows pre-processed reference spectra of backgrounds and single-layer plastic materials used, which are helpful for further analysis of influences in the loading plots.

The impact of various background materials on the sample spectra was evaluated by comparing the loading plots to the reference spectra of the backgrounds, as illustrated in Figure 5a. Additionally, each wavelength where significant spectral effects, either from the backgrounds or different materials within the multilayer films, were detected are numerically indicated for clarity, and these numbers are correspondingly mentioned in the text alongside the associated spectral peaks. The influence of white tile can first be detected in PC3 at the wavelength range of 2200–2300 nm (Figure 5, wavelengths *1* and *2*), while the Teflon can already be detected in PC1 at 1680 (Figure 5, wavelength *3*), where the loading plots of Teflon shows different behavior than the loading plots of samples measured with different backgrounds.

All metallic backgrounds show peaks in the same wavelengths but with different intensities. The metallic backgrounds are most visible in PC3 at wavelength ranges of 1600–1800 nm (Figure 5, wavelengths *4* and *5*). The peaks are pronounced differently, with higher or lower intensities depending on the different metallic backgrounds used. Although the behavior of the metallic backgrounds can be recognized, no strongly pronounced peaks are to be expected to influence further classification. At the same time, the same cannot be stated for Teflon since its influence has already been detected in PC1 and may affect further identification.

The differences between PO and NPO spectra can be analyzed using loading plots, which are compared with the reference spectra of monolayer materials in Figure 5b. It can be seen that the behavior of PE is often recognizable in the loading plots, particularly in the wavelength range of 1750 (*1*), 2300 (*2*), and 2330 nm (*3*) and also already in the PC1, but also in the other PCs with different intensities. In contrast, PP's spectral behavior can be seen above all in the peak at 1690 nm (*4*), with the most pronounced intensity in the PC2. The characteristic peaks of the plastic types not belonging to the polyolefin group can also be recognized. PET can be recognized already in the PC1 at 2290 nm (*6*) and at 1680 nm (*5*) in PC3. The characteristic peak of PA at 2050 nm (*7*) is recognizable in both PC2 and PC3 but not in PC1, which would explain the grouping of PET-PE and PE-PA NPO fractions on one side of the score plots compared to the PET-PP fraction of NPO on the other side of the score plots. It is worth highlighting that PET and PA's characteristic peaks are more pronounced when measured using metallic backgrounds, compared to the tile and Teflon, which indicated the higher possibility of correct classification when using metallic backgrounds.

It can be concluded that PE is most strongly represented, corresponding to the fact that it is mainly found in the plastic material within multilayer fraction, which shows the significant contribution to the spectra of multilayer films. Since PE is present in both PO and NPO fraction samples, there is the risk of identifying the NPO samples as PO, eliciting possible false positive results. However, these concerns can be refuted because the PE and PP are still strongly represented, showing the probability of them being able to be separated from the other multilayer fractions.

### K-Nearest Neighbors (KNN) Classification

**Figure 6.**
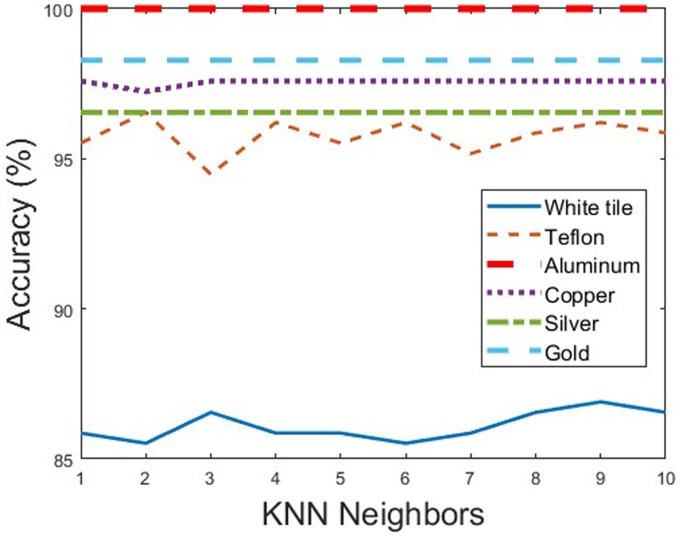
KNN identification rate.

The classification accuracy of the KNN classification was performed in Matlab, following the application of PCA, using a test set of samples. Multiple classification methods were applied to each scenario for comparison to analyze the impact of varying numbers of neighbors and different measurement backgrounds on classification. The results showed no significant difference, especially for the metallic backgrounds where the classification remains consistent, demonstrating the stability of the spectra when acquired with metallic backgrounds. The results are shown graphically in Figure 6, where it can be seen that the metallic backgrounds (aluminum, copper, silver and gold) have higher and constant levels of accuracy compared to the non-metallic backgrounds (white tile, Teflon), which is consistent with the analyzed results of PCA. The results represent theoretical results that provide sufficient certainty to perform a classification with a handheld NIR spectrometer to obtain experimental results.

### Classification Using a Handheld NIR Spectrometer

The methods for performing the experimentally correct classification using Method Generator software were developed upon reaching successful outcomes of the theoretically possible classification. Since using different neighbors in the theoretical KNN classification performed in Matlab did not result in significant differences for most backgrounds, the KNN classification in the Method Generator Software is performed with one neighbor. Using the developed methods, the classification with a handheld NIR spectrometer was performed using a test set of samples, always using the same measuring background as in the developed method. Each sample from the test set was measured five times using the developed methods to ensure comparability with theoretically possible classification, which utilized five spectra per sample for the analysis. The repeated measurements did not influence the achieved accuracy for any of the developed methods, except for Teflon, where the presented accuracy is the mean accuracy of the repeated measurements. However, they enhanced the reliability of the experimentally correct classification results. Table 1 shows the accuracy of the classifications performed with the developed methods with all the backgrounds used. The results show high accuracy of experimentally correct classification for all backgrounds, but with compared lower accuracy when using Teflon.

**Table 1.** Accuracy of the test set of samples analyzed using NIR handheld spectrometer.

	WHITE TILE	TEFLON	ALUMINUM	COPPER	SILVER	GOLD
Accuracy (%)	100	72.22	100	100	100	100

## Conclusion

A handheld NIR spectrometer in the NIR region of 1596–2396 nm was examined to classify multilayer polyolefin films and the influence of different measuring backgrounds on the classification’s accuracy. The results showed that handheld NIR spectrometers are reliable for fast and robust on-site classification of multilayer polyolefin films. Identifying multilayer film compositions measured from only one side of the sample is particularly advantageous.

The method was initially developed using a white tile as a measuring background. Solid results showed that polyolefin multilayer films can be identified and classified with 86.90% theoretical and 100% experimental accuracy. The tiles can often be glazed using titanium dioxide (TiO_2_), which has a maximum reflectivity of up to 0.9 at the IR wavelength. This may explain the high experimental accuracy when measuring with the tile as background. However, the results’ lower theoretical accuracy and lower constancy still show possible disadvantages when using the tiles. Since the low material thickness of films imposed a challenge because they provided lower spectral quality, various metallic backgrounds, including aluminum, copper, silver, and gold, were used and tested to improve the spectral quality and classification accuracy. The influence of the color of different samples was not further observed in this study, since it was recognised that the color of each sample does not significantly affect the classification accuracy as long as the sample has not been colored with carbon black.

The use of metallic backgrounds increased the spectral quality of measured samples, resulting in higher classification accuracy. The theoretically possible classification using KNN classification resulted in very high accuracy with slight deviations between the metallic backgrounds (96.55–100%), with aluminum and gold backgrounds providing the best results.

Conversely, the experimental classification showed an accuracy of 100% when measured with all metallic backgrounds, meaning that the classification accuracy reaches a high level when metallic backgrounds are used. The material Teflon was also tested, which showed a solid theoretical accuracy of 96.21% but a low experimental accuracy of only 72.2%. The discrepancy between the theoretical and experimental results of Teflon can be attributed to the treatments and modifications applied to each application of Teflon. The Teflon used for the measurements was the one used for laboratory microwave measurements and may not have undergone the same surface treatment as Teflon used as a highly reflective surface. It is also common for theoretical accuracy to surpass experimental accuracy due to various factors that can influence data collection. Notably, the disparity in obtaining theoretical and experimental accuracy arises from the fact that the theoretical accuracy was derived solely through the use of Matlab. In contrast, the experimental accuracy was directly obtained by testing the methods developed for the handheld NIR spectrometer in the associated software.

Thus, using metallic backgrounds when performing measurements using a handheld NIR spectrometer to measure plastic materials provides promising results for improving spectral quality and classifying plastic materials with low thickness (films) and complex material composition (multilayers). This work provides a basis for further developing the identification and classification of challenging plastic material fractions using a handheld NIR spectrometer. Further perspectives can be seen in the possibility of developing additional methods for the fast and robust identification and classification of other multilayer fractions using a handheld NIR spectrometer.
